# Relapse Rates in Patients with Multiple Sclerosis Switching from Interferon to Fingolimod or Glatiramer Acetate: A US Claims Database Study

**DOI:** 10.1371/journal.pone.0088472

**Published:** 2014-02-06

**Authors:** Niklas Bergvall, Charles Makin, Raquel Lahoz, Neetu Agashivala, Ashish Pradhan, Gorana Capkun, Allison A. Petrilla, Swapna U. Karkare, Catherine Balderston McGuiness, Jonathan R. Korn

**Affiliations:** 1 Novartis Pharma AG, Basel, Switzerland; 2 IMS Health, Plymouth Meeting, Pennsylvania, United States of America; 3 Novartis Pharmaceuticals Corporation, East Hanover, New Jersey, United States of America; Charité University Medicine Berlin, Germany

## Abstract

**Background:**

Approximately one-third of patients with multiple sclerosis (MS) are unresponsive to, or intolerant of, interferon (IFN) therapy, prompting a switch to other disease-modifying therapies. Clinical outcomes of switching therapy are unknown. This retrospective study assessed differences in relapse rates among patients with MS switching from IFN to fingolimod or glatiramer acetate (GA) in a real-world setting.

**Methods:**

US administrative claims data from the PharMetrics Plus™ database were used to identify patients with MS who switched from IFN to fingolimod or GA between October 1, 2010 and March 31, 2012. Patients were matched 1∶1 using propensity scores within strata (number of pre-index relapses) on demographic (e.g. age and gender) and disease (e.g. timing of pre-index relapse, comorbidities and symptoms) characteristics. A claims-based algorithm was used to identify relapses while patients were persistent with therapy over 360 days post-switch. Differences in both the probability of experiencing a relapse and the annualized relapse rate (ARR) while persistent with therapy were assessed.

**Results:**

The matched sample population contained 264 patients (n = 132 in each cohort). Before switching, 33.3% of patients in both cohorts had experienced at least one relapse. During the post-index persistence period, the proportion of patients with at least one relapse was lower in the fingolimod cohort (12.9%) than in the GA cohort (25.0%), and ARRs were lower with fingolimod (0.19) than with GA (0.51). Patients treated with fingolimod had a 59% lower probability of relapse (odds ratio, 0.41; 95% confidence interval [CI], 0.21–0.80; *p = *0.0091) and 62% fewer relapses per year (rate ratio, 0.38; 95% CI, 0.21–0.68; *p = *0.0013) compared with those treated with GA.

**Conclusions:**

In a real-world setting, patients with MS who switched from IFNs to fingolimod were significantly less likely to experience relapses than those who switched to GA.

## Introduction

Multiple sclerosis (MS) is a chronic inflammatory, immune-mediated disease of the central nervous system [1], [2] that affects approximately 400,000 people in the USA and 2.1 million people worldwide [3]. Relapsing–remitting MS (RRMS) is the most common type of MS, affecting approximately 80–85% of all patients with MS [4], and is characterized by unpredictable acute attacks (known as relapses) accompanied by worsening of symptoms, followed by periods of remission during which there is a full or partial recovery from the deficits acquired during the relapse. Relapse activity is associated with an increased risk of disability progression [5], [6], although disability can advance independently of relapse activity (secondary progressive MS) [7]. Treatments for MS traditionally aim to modify the disease by reducing the number and severity of relapses and delaying the progression of disability. Modern therapeutics aim to keep patients free of disease activity (relapses, disability progression or MRI activity). For more than two decades, disease-modifying therapies (DMTs) such as interferons (IFNs) and glatiramer acetate (GA) have been used for the first-line treatment of patients with RRMS [8], [9], [10]. These immunomodulatory agents have a comparable degree of efficacy in MS; the different IFN formulations are generally considered to have similar efficacy [11], and two large direct comparative studies have demonstrated that IFN and GA are also similar in their efficacy [12], [13]. However, for many patients with MS, the effectiveness of these DMTs is relatively low, and their tolerability profiles are considered suboptimal [14].

Some patients may need to switch from one DMT to another owing to treatment-related issues such as unresponsiveness (i.e. disease progression) or intolerance. Injection-site reactions are the most commonly reported side effects of non-oral DMTs [14], [15]. IFNs are associated with influenza-like symptoms, which are experienced by 75% of patients, and there are also concerns that IFNs may cause or worsen depression [14]. IFNs are the most commonly prescribed DMTs for MS in the USA [16], with a reported market share of approximately 46% in October 2012 [17]. However, one-third of patients treated with IFNs are reported to be unresponsive to treatment (defined as having had more than one relapse or a sustained Expanded Disability Status Scale [EDSS] score increase of ≥0.5 points after 1 year of treatment compared with the year prior to therapy) [18]. Relapses are considered to be an important measure of treatment response because they have been found to be an important predictor for future development of disability [19]. In addition, a review of discontinuation rates across several countries found that 16–27% of patients had been reported to discontinue IFN therapy prematurely over the short term, which increases to 43% when patients were followed longer than 24 months [20]. Unresponsiveness may in part reflect poor adherence to medication [21], [22].

At present, there is limited real-world information regarding which therapy provides the best clinical response in patients with RRMS following a switch. In the phase 3, 12-month Trial Assessing Injectable Interferon versus FTY720 Oral in Relapsing–Remitting Multiple Sclerosis (TRANSFORMS), fingolimod, the first oral therapy approved for the treatment of relapsing MS, demonstrated a significant reduction in annualized relapse rate (ARR) compared with intramuscular IFN beta-1a (ARR was 0.16 in the fingolimod group compared with 0.33 in patients treated with IFN; *p*<0.001) [23]. In the extension of TRANSFORMS, patients who received IFN in the core study had a significant reduction in ARR within 1 year of switching to fingolimod therapy [24]. Additional clinical benefits relative to injectable DMTs may also be gained from the once-daily, oral administration of fingolimod, which is well tolerated [25], [26], potentially resulting in increased adherence to therapy. The phase 4 EPOC (Evaluate Patient Outcomes, Tolerability, and Safety of Fingolimod) study described increased patient-reported treatment satisfaction and physician-assessed clinical improvements for patients switching to fingolimod compared with those remaining on IFN or GA [27], [28]. A recent database analysis has demonstrated that patients with MS initiating fingolimod therapy had higher rates of persistence and adherence than patients using injectable DMTs [29], [30]. Rates of persistence over 12 months were 74.3% for fingolimod, 42.9–54.1% for IFN and 62.6% for GA in patients who had previously used DMTs [29]. Our study, a retrospective cohort analysis of a US health insurance claims database, was performed to assess differences in relapse rates among patients with MS who switched from IFN to fingolimod and those who switched from IFN to GA, in a real-world setting.

## Materials and Methods

### Data Source

This study was a retrospective cohort analysis of the PharMetrics Plus™ claims database, which contains adjudicated medical and pharmacy claims for more than 87 million health plan members across the USA from 2006 onwards. The data are longitudinal, with approximately 22 million patients having 4 or more years of continuous enrollment in their health plan. The database is representative of the US commercially insured population and has broad geographical coverage, including patients in each three-digit zip code area of the USA and data from 90% of US hospitals and 80% of all US doctors, and from employees in 85% of the Fortune 100 companies. The database specifically includes integrated claims data (i.e. data from multiple places of service [inpatient, outpatient and pharmacy]) for over 100,000 patients with MS in 2011 and is therefore believed to be representative of the population of patients with MS in the USA.

Within the database, inpatient and outpatient diagnoses were recorded as International Classification of Diseases, Ninth Revision, Clinical Modification (ICD-9-CM) codes; other data recorded included inpatient and outpatient procedures, dates of service, retail and mail-order prescription records, and detailed information on pharmacy and medical benefit (co-payment/coinsurance amount, deductible, and in-network versus out-of-network), inpatient stay (admission versus that for other diagnoses, admission type and source, and discharge status) and provider details (specialty, zip code and attending, referring, rendering, prescribing and primary care provider). Detailed ICD-9-CM diagnosis and procedural codes were available for each hospital stay; however, the names and descriptions of specific medications used during inpatient stays were not available. It was therefore not possible to identify the use of any DMT during a hospital stay, although DMTs administered in an outpatient clinic or infusion center setting could have been be identified, provided that the treatment did not require an overnight hospital stay. Amounts charged by providers, and amounts allowed and paid by health plans, were available for all services rendered, as were the dates of service for all claims. Other data elements included demographic variables (patient age, gender and geographical region), product type (e.g. health maintenance and preferred provider organizations), payer type (e.g. commercial, self-pay) and start and stop dates of health-plan enrollment.

### Ethics Statement

The PharMetrics Plus™ database is fully compliant with the Health Insurance Portability and Accountability Act of 1996 (HIPAA) privacy regulations. Access to the PharMetrics database requires a licensing agreement and the data are provided de-identified. Open access to the data used in this study is not permitted under the data licensing agreement. As all patient-level data are HIPAA-compliant and certified anonymous, Institutional Review Board approval and patient informed consent were not required for this study. This study was designed, implemented and reported in accordance with the Guidelines for Good Pharmacoepidemiology Practices (GPP) of the International Society for Pharmacoepidemiology, the Strengthening the Reporting of Observational Studies in Epidemiology (STROBE) guidelines [31], and with the ethical principles laid down in the Declaration of Helsinki.

### Patient Selection

**Figure 1:**
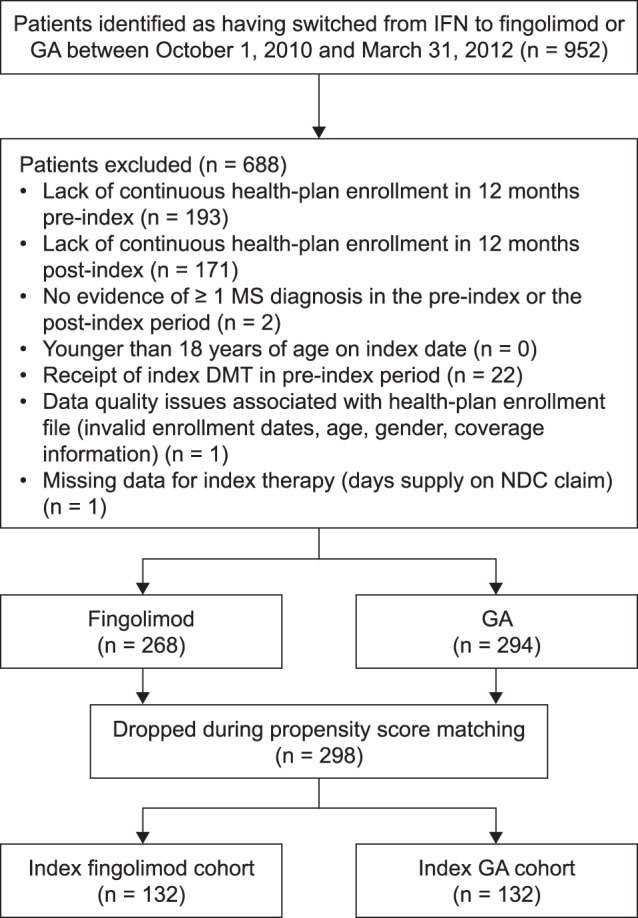
Attrition of the study sample, by reason. Abbreviations: DMT, disease-modifying therapy; GA, glatiramer acetate; IFN, interferon; MS, multiple sclerosis; NDC, National Drug Code. ^a^Patients were propensity-score matched within strata (number of pre-index relapses) on age, gender, region, health-plan type, prescribing physician specialty, Charlson comorbidity index score, pre-index use of dalfampridine, relapse within 90 days pre-index, pre-index total costs, symptoms (numbness, fatigue and bowel symptoms) and comorbidities (depression and diabetes mellitus).

Patients with a diagnosis of MS (ICD-9-CM code 340) who had switched from IFN therapy (IFN beta-1a [intramuscular Avonex® or subcutaneous Rebif®] or IFN beta-1b [Extavia® or Betaseron®, both administered subcutaneously]) to fingolimod (Gilenya®, administered orally) or GA (Copaxone®, administered subcutaneously) between October 1, 2010 and March 31, 2012 (index window) were identified in the database (National Drug Codes [NDCs] used are listed in Table S1 and procedural codes for DMTs administered in the clinical setting are listed in Table S2). The first observed medication switch date was defined as the index date, and this was the only switch assessed. Medical and pharmacy records for eligible patients were then studied for 360 days following the index date. Patients were included if all of the following criteria were met: evidence of a medication switch from IFN therapy to fingolimod or GA (with initiation of fingolimod or GA occurring within 90 days of a claim for IFN); continuous health-plan enrollment for a minimum of 360 days before and after the index date (the pre- and post-index periods, respectively); at least one claim with an MS diagnosis within 360 days of the index date (pre- or post-index); and aged 18 years or older on the index date. Patients were excluded from the analysis if they had received their index DMT (fingolimod or GA) in the pre-index period, had a gap in the claims data indicative of missing days supply information for the index therapy or had data quality issues (e.g. invalid enrollment date, incomplete claims data, missing or invalid age and/or gender; Figure 1).

### Propensity Score Matching

Patients receiving fingolimod were randomly matched to patients receiving GA using propensity score methodology [32]. Propensity scores were calculated for each patient and represent their probability of receiving a given treatment based on baseline characteristics. Scores were calculated by summing coefficient values for a list of potential confounding baseline variables. Use of these scores allows a single estimate to be employed to adjust for baseline imbalances, and enables patients on different treatments to be matched taking multiple variables into account simultaneously.

Propensity scores were derived from a logistic regression model, in which patients receiving fingolimod were matched with those receiving GA within each stratum (the number of relapses occurring in the pre-index period). Use of fingolimod therapy was the dependent variable and pre-index characteristics, which were entered in a stepwise fashion into the logistic regression model, were the independent variables. Final independent variables were: age, gender, region, health-plan type, prescribing physician specialty, Charlson comorbidity index score [33], [34], pre-index use of dalfampridine, relapse within 90 days pre-index, pre-index total costs, symptoms (numbness, fatigue and bowel symptoms) and comorbidities (depression and diabetes mellitus). Patients from each treatment cohort were matched on a 1∶1 basis using the ‘nearest neighbor’ approach, to identify pairs of patients based on similarity of propensity scores and discarding the cases defined by a minimal difference (e.g. ±0.01) in the fitted probability of DMT use.

### Study Measures

The primary measure of interest was the number of relapse events experienced in the 360-day post-index period. Currently, there are no specific diagnosis codes or procedural codes with which to identify MS relapses in medical claims databases directly. Relapses were therefore defined using an algorithm based on one tested in several previous analyses of claims data [35], [36].

Inpatient relapses were defined as relapses requiring an inpatient visit with a primary ICD-9-CM diagnosis code of 340. Outpatient relapses were defined as relapses requiring an outpatient visit with a diagnosis code of 340 and oral or intravenous corticosteroid use within 7 days of the outpatient visit [37]. Events without a primary exclusionary diagnosis code for oral or intravenous corticosteroids (including asthma, gout, rheumatoid arthritis and uveitis) on the date of the outpatient visit were included. Relapse events occurring within the same 30-day period were classified as a single event. Relapses were measured in the pre- and post-index periods for all patients, as well as while patients were persistent (a measure of how long patients remain on therapy) with fingolimod or GA. In this study, persistence was measured based on treatment patterns (i.e. number of consecutive days from initiation of index DMT monotherapy until discontinuation, receipt of another DMT of interest [IFN, GA, fingolimod or natalizumab] or the end of the available data period [360 days post-index], whichever occurred first). In line with previous studies that evaluated persistence in MS, discontinuation was defined as a gap in exposure to the index DMT of at least 60 days following the date on which the index DMT should next have been dispensed or administered [29], [38]. The proportion of patients experiencing a relapse, the total number of relapses observed during the post-index period and ARRs were evaluated for both treatment cohorts. Other data recorded included patient demographics and baseline characteristics, which were evaluated during the pre-index period. These included age, gender, previous use of dalfampridine, Charlson comorbidity index score, comorbidities of interest (e.g. dyslipidemia, depression), occurrence of any MS relapse and number of MS relapses.

### Statistical Analyses

For categorical measures, data are presented as counts and proportions. Continuous variables were summarized by providing the mean, 95% confidence interval (CI), standard deviation (SD) and median. Differences in the distribution of these variables were tested for statistical significance using chi-square tests for categorical variables and the Wilcoxon rank-sum test for continuous variables. A logistic regression model was used to estimate the probability of experiencing a relapse while persistent with the index medication. The dependent variable was the presence of a relapse while persistent with therapy and the offset variable was the log of the number of years on therapy. Differences in the number of relapses (ARRs) while persistent with the index medication were estimated using a negative binomial regression model; the number of relapses served as the dependent variable and the log of the number of years on therapy was the offset variable. Given the matched nature of the data, all generalized linear models were fitted with generalized estimating equations (GEEs).

Time to relapse (in days) while persistent with the index medication was described using Kaplan–Meier analysis, with separate survival curves for each cohort. The probability of experiencing a relapse over time was calculated based on the number of patients still being followed through the post-index period. Patients were followed until relapse, discontinuation of index therapy or the end of the available data period (360 days post-index), whichever occurred first. Statistical significance of the differences between curves was assessed using the log-rank test.

### Sensitivity Analyses

Sensitivity analyses of regression models were performed to adjust for baseline symptoms that were not included in the propensity score matching procedure and that affected more than 5% of patients in the total sample: headache, muscle weakness/spasms/spasticity, visual symptoms, bladder dysfunction, dizziness and vertigo, respiration/breathing problems, and problems with walking, balance and coordination (fatigue, numbness and bowel dysfunction were included in the matching).

## Results

### Study Attrition

In total, 952 patients were initially identified as having switched from IFN to fingolimod or GA in the index window, of whom 688 (72.3%) were excluded for the reasons listed in Figure 1. A total of 132 patients were therefore included in each of the fingolimod and GA cohorts.

### Pre-index Demographics and Clinical Characteristics

The pre-index demographics and clinical characteristics of the matched fingolimod and GA switch cohorts are shown in Table 1. Three-quarters of the patients included in the study were female and the median ages of patients were similar between cohorts (47 and 46 years for fingolimod and GA, respectively; *p* = 0.5131). Some symptoms were more common in the GA cohort than the fingolimod cohort (e.g. headache, muscle weakness/spasm/spasticity and visual symptoms), although no significant differences were reported between groups. The prevalence of comorbidities (e.g. diabetes mellitus and dyslipdemia) was similar between cohorts.

**Table 1:** Demographic and clinical characteristics of patients in the fingolimod and GA cohorts in the pre-index period.

Characteristic	Fingolimod (n = 132)	GA (n = 132)	*p* value
Age, years			
Mean ± SD	46.1±10.4	45.5±9.9	
Median	47.0	46.0	0.5131
Female, n (%)	96 (72.7%)	102 (77.3%)	
Previous use of dalfampridine, n (%)	12 (9.1%)	9 (6.8%)	0.4950
Charlson comorbidity index score, mean ± SD	0.48±0.91	0.43±0.84	
Symptoms affecting ≥10% of patients, n (%)			
Fatigue	45 (34.1%)	43 (32.6%)	0.7940
Walking (gait), balance, and coordination problems	26 (19.7%)	22 (16.7%)	0.5233
Numbness	25 (18.9%)	27 (20.5%)	0.7569
Headache	22 (16.7%)	31 (23.5%)	0.1667
Muscle weakness/spasm/spasticity	16 (12.1%)	21 (15.9%)	0.3754
Visual symptoms	15 (11.4%)	23 (17.4%)	0.1607
Bladder dysfunction	14 (10.6%)	13 (9.8%)	0.8390
Comorbidities affecting ≥5% of patients, n (%)			
Dyslipidemia	35 (26.5%)	34 (25.8%)	0.8886
Depression	33 (25.0%)	29 (22.0%)	0.5614
Tobacco use (including disorder)	10 (7.6%)	8 (6.1%)	0.6253
Diabetes mellitus	8 (6.1%)	11 (8.3%)	0.4750
History of CVD	8 (6.1%)	8 (6.1%)	1.0000
No. of pre-index relapses, mean ± SD	0.46±0.79	0.49±0.90	
Patients experiencing relapses in the pre-index period, n (%)	44 (33.3%)	44 (33.3%)	1.0000
0 relapses	88 (66.7%)	88 (66.7%)	0.9805
1 relapse	33 (25.0%)	33 (25.0%)	
2 relapses	6 (4.5%)	5 (3.8%)	
≥3 relapses	5 (3.8%)	6 (4.5%)	
Patients experiencing an outpatient relapse in the pre-index period,^a^ n (%)	39 (88.6%)	44 (100.0%)	
Patients experiencing an inpatient relapse in the pre-index period,^a^ n (%)	6 (13.6%)	2 (4.5%)	
Healthcare costs, US$			
Total, mean ± SD	41,972±17,986	40,753±15,884	
Median	40,050	40,150	0.7704

CVD, cardiovascular disease; GA, glatiramer acetate; SD, standard deviation.

a Among those patients who had a relapse; percentages do not add up to 100% as some patients experienced both inpatient and outpatient visits.

During the pre-index period, one-third of patients in both cohorts had at least one relapse. All such patients in the GA cohort experienced an outpatient relapse during this period compared with 89% of the fingolimod cohort, although more patients experienced inpatient relapses in the fingolimod cohort compared with the GA cohort (13.6% and 4.5%, respectively). As expected after the propensity score matching, ARRs were similar in both cohorts during the 360-day pre-index period (fingolimod: 0.46, GA: 0.49).

### Persistence with Fingolimod and GA after Switching From IFN Therapy

The proportion of patients who were persistent with medication during the post-index period was higher among those who switched to fingolimod than among those who switched to GA (73.5% versus 62.9%) although the difference was not statistically significant (*p* = 0.0643). The mean ± SD persistence period was longer for the fingolimod cohort than the GA cohort (294±118 days and 272±126 days, respectively).

### Proportion of Patients with Relapses in the Fingolimod and GA Switch Cohorts

**Figure 2:**
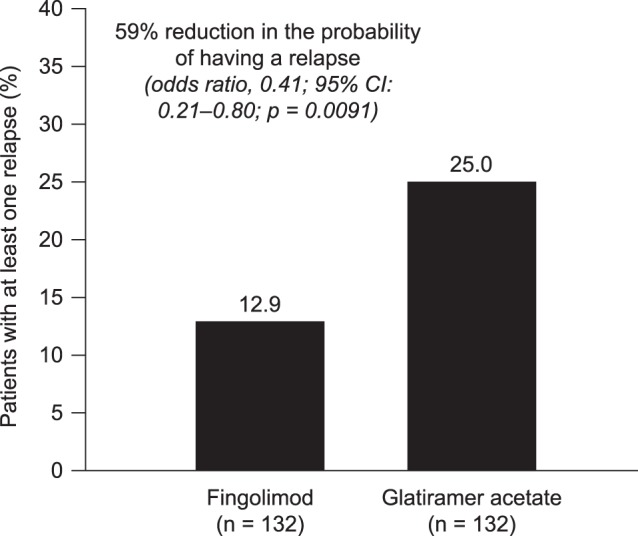
Proportions of patients with at least one relapse during the post-index persistence period. CI, confidence interval.

**Figure 3:**
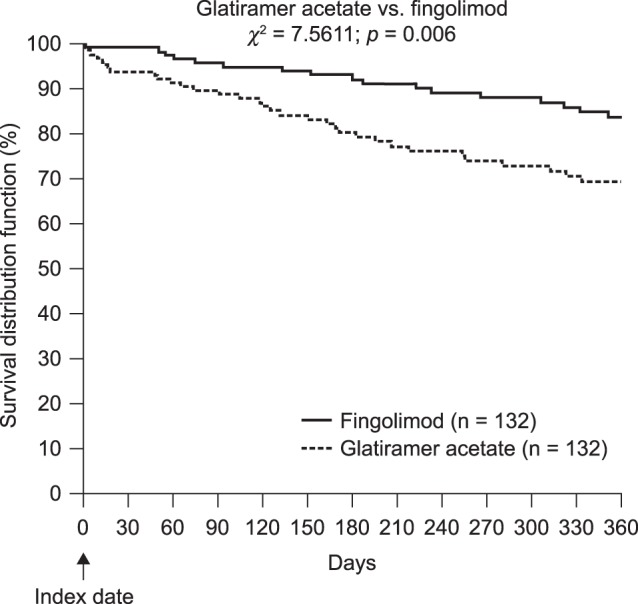
Time to relapse while persistent with therapy (Kaplan–Meier analysis).

The proportion of patients with at least one relapse in the post-index persistence period was significantly lower in the fingolimod cohort than in the GA cohort (12.9% and 25.0%, respectively, *p* = 0.0120; Figure 2). During the post-index persistence period, fingolimod was associated with a 59% reduction in the probability of having a relapse compared with GA (odds ratio [OR], 0.41; 95% CI, 0.21–0.80; *p* = 0.0091). In sensitivity analyses, in which symptoms not included in the matching procedure were included as independent variables, the corresponding reduction was 63% (OR, 0.37; 95% CI, 0.18–0.77). The median time to first relapse was 360 days for the fingolimod cohort and 274 days for the GA cohort. In addition, time to first relapse while persistent with medication was significantly longer in the fingolimod group than in the GA group (χ^2^ test for GA versus fingolimod: 7.56, *p* = 0.006; Figure 3).

### Relapse Rates in the Fingolimod and GA Switch Cohorts

**Figure 4:**
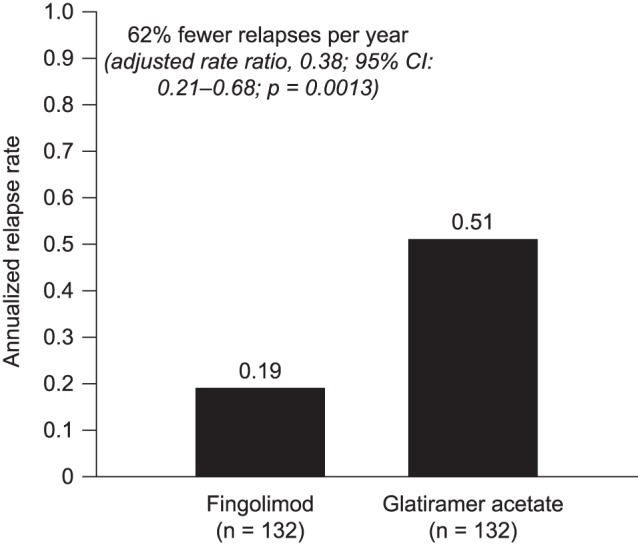
Relapse rates during the post-index persistence period. CI, confidence interval. Annualized relapse rates were based on generalized estimating equations regression using a negative binomial distribution.

Based on the negative binomial regression, ARRs during the post-index persistence period were lower in the fingolimod cohort than in the GA cohort (0.19 and 0.51, respectively; Figure 4). Patients treated with fingolimod had 62% fewer relapses per year (rate ratio [RR], 0.38; 95% CI, 0.21–0.68; *p* = 0.0013) during this period than those who switched to GA. In sensitivity analyses, in which symptoms not included in the matching procedure were included as independent variables, the corresponding reduction was 61% (RR, 0.39; 95% CI, 0.22–0.71).

## Discussion

Few studies have investigated the clinical outcomes of switching DMTs in patients with MS in a real-world setting. To our knowledge, this is the first retrospective US claims database analysis to assess relapse rates among patients with MS who switched from IFN to either fingolimod or GA. Our study demonstrates that relapse rates were significantly lower in patients who switched to fingolimod than in those who switched to GA. In addition, fewer patients who switched to fingolimod had relapses than patients who switched to GA.

As the cohorts were matched by propensity score, there was no difference between the fingolimod and GA cohorts in the proportion of patients experiencing at least one relapse (33.3% for both groups) or the ARR (0.46 and 0.49, respectively) in the pre-index period. In contrast, the proportion of patients experiencing a relapse while persistent with medication in the post-index period was lower for fingolimod than for GA (12.9% and 25.0%, respectively) as was the ARR (0.19 and 0.51, respectively). Patients treated with fingolimod had a 59% lower probability of experiencing a relapse, 62% fewer relapses per year and a longer time to relapse (*p* = 0.006) than patients treated with GA. Results of sensitivity analyses adjusting for baseline differences in symptoms (not included in the matching procedure) between the cohorts were similar to those in the main analysis. Taken together, these data indicate that fingolimod is more effective than GA at reducing relapses in patients switching from IFN therapy.

These analyses confirm the results from the pivotal clinical trials with fingolimod. Clinical outcomes of switching from IFN therapy to fingolimod have been assessed in the 12-month extension of TRANSFORMS, in which relapse rates were compared in patients who switched from IFN to fingolimod at baseline or after 1 year [24]. Patients switching after 1 year had a significantly reduced ARR during fingolimod treatment compared with IFN treatment in year 1 (0.22 and 0.31, respectively; *p* = 0.049), which is similar to the post-switching ARR of 0.19 reported in the present study. A post-index ARR of 0.51 was observed for patients switching to GA in the present study, which is very similar to an ARR of 0.53 reported in a prospective study of 85 patients in the USA who switched from IFN to GA because of lack of efficacy or intolerance [39], and broadly comparable with ARRs of 0.34–0.81 reported in four prospective, randomized clinical trials for GA [13], [40], [41], [42]. In the present study, relapses were identified using a claims-based algorithm rather than a clinical examination by a neurologist as in the clinical trials. Although the algorithm may be a less sensitive way of detecting relapses than methods used in the clinical trials, similarities in the ARRs reported using these different techniques support the use of these algorithms for assessing relapses in administrative claims databases.

The occurrence of relapses in patients taking GA has previously been assessed in two retrospective cohort claims database studies. In a study of 4334 patients with MS treated with GA with or without antihistamine, 10.9% of patients had at least one relapse during the follow-up period versus 25.0% in the present study, although patients did not switch therapy in that study and were only followed for 10 months [43]. In a second study of 2446 individuals with MS, 31.1% of patients had relapses while adherent to therapy, although only 35.4% of patients were receiving GA and the rest were taking IFN [22]. No head-to-head trials have assessed the comparative efficacy of fingolimod and GA, but a mixed-treatment comparison used in a meta-analysis of prospective, comparative clinical trial data for various DMTs in RRMS found that fingolimod was associated with a 30% reduction in ARR compared with GA [44]. These limited data support the findings of the present study and suggest that fingolimod is more effective than GA at controlling relapses.

Choosing an MS treatment that is optimal for the patient is challenging for both patients and physicians. Current treatment guidelines (developed before the availability of newer agents such as fingolimod) recommend that, on diagnosis of MS, therapy is initiated and maintained with one of the three first-line approved IFNs or GA [45]. However, these therapies may have limited effectiveness and are associated with adverse events that may lead to patients needing to switch to another DMT [15]. Switching therapy is known to incur higher costs, with a recent large-scale study reporting that non-pharmacy medical costs were significantly increased by 50% in patients who switched from first-line DMTs compared with those who remained persistent with therapy [15]. In addition, there is a period of time before some DMTs become fully effective (for example, GA may take up to 9 months to become fully effective [46]), so it is possible that patients may lose disease control in the first few months after switching therapy. For fingolimod, on the other hand, it has been shown that initial treatment effects are already present after approximately 3 months [47]. In the present study, it is possible that, for most of the 1-year follow-up, GA was not yet fully effective in patients, and the higher relapse rates seen in the GA cohort may reflect loss of disease control as a result.

Highly effective and rapid disease control combined with a tolerable safety profile and administration are critical aspects of MS treatment. There is emerging evidence that patients treated with the well-tolerated, oral DMT fingolimod are significantly more likely to be adherent to treatment and less likely to discontinue their medication than those treated with injectable DMTs [29]. Additional research is needed to evaluate the association between a break in disease control and an increase in healthcare costs. There may be an additional clinical benefit to switching early. The TRANSFORMS extension found that patients treated with fingolimod from baseline (the majority of patients in TRANSFORMS had received previous treatment with IFN or GA) had a lower ARR in year 2 than those who switched after 1 year of IFN therapy (0.18 and 0.22, respectively) [24], and that this effect is also seen after 4.5 years. [48] As such, it is likely that switching earlier will confer additional benefits to patients. The tolerability profile of fingolimod additionally leads to the expectation that adherence to fingolimod would be better than that to other currently available DMTs, including IFNs and GA; this would reduce the need for switching, with the associated break in disease control and increase in healthcare costs. This expectation is supported by a previous US claims database analysis, which reported that patients treated with fingolimod were significantly more likely to be adherent than patients treated with injectable DMTs [29]. The same study also demonstrated that patients in whom fingolimod therapy was initiated were less likely to discontinue treatment, and those who discontinued did so later than patients using injectable DMTs [49].

A strength of this study was that data were derived from a large US administrative health-plan database, which contains more than 150 million adjudicated claims, including inpatient, outpatient and pharmacy data from multiple payers, and is considered to be representative of the US commercially insured population. Such data provide an excellent resource for assessing treatment patterns and outcomes in a real-world setting. The database also contains information on over 100,000 patients with MS and provides insights into clinical outcomes for patients being treated with GA and fingolimod, which are limited in the literature at present. Nevertheless, retrospective database analyses are subject to some limitations, against which the present findings must be considered. The results are based on medical and pharmacy claims and do not provide information on whether medications were used as prescribed. In addition, diagnoses can be miscoded, and chart review and verification of data were not possible. However, for inclusion of patients, our study required both a diagnosis of MS and a prescription for a DMT, reducing the likelihood of including non-MS patients. Furthermore, the algorithm for defining relapses was partially based around treatments received, the criteria for which vary considerably among physicians. However, the algorithm used is based on one used in several previous database claims analyses [35], [36], and the results obtained in this study are similar to those from prospective controlled studies, supporting the validity of the approach. Another possible limitation of the study is that clinical measures, such as MS severity and progression and lesion type data, were not readily available in the claims database. Such endpoints would have provided additional detail and insight into treatment patterns reported in this study. Finally, the analytic focus was on patients who met continuous enrollment criteria (12 months pre- and post-index therapy), which may have excluded patients with different treatment patterns. This type of continuous enrollment restriction was necessary to ensure that full details of patients’ treatment were captured in the pre- and post-index periods, and to allow comparison between cohorts.

In conclusion, in a real-world setting, patients who switched from IFN to fingolimod were significantly less likely to experience relapses than those who switched from IFN to GA. As relapse rates impact on disability progression and health-related quality of life, these findings suggest that more favorable long-term outcomes are likely to be achieved in patients who switch from IFN to fingolimod as opposed to GA. This study provides valuables insight into the real-world outcomes of treatments for MS.

## Supporting Information

Table S1 **NDCs for DMTs of interest.** DMT, disease-modifying therapy; GA, glatiramer acetate; HCl, hydrochloride; IFN, interferon; i.m. intramuscular; i.v. intravenous; NDC, National Drug Code; s.c. subcutaneous.
(DOCX)

Table S2 **Procedural codes for DMTs administered in the clinical setting.** DMT, disease-modifying therapy; GA, glatiramer acetate; IFN, interferon; N/A, not applicable.
(DOCX)
