# Supplementary material for: Relapse Rates in Patients with Multiple Sclerosis Switching from Interferon to Fingolimod or Glatiramer Acetate: A US Claims Database Study
Source: PLoS One. 2014 Feb 6;9(2):e88472. doi: 10.1371/journal.pone.0088472 (PMC3916439; doi:10.1371/journal.pone.0088472)
Supplement: Table S1 — NDCs for DMTs of interest. DMT, disease-modifying therapy; GA, glatiramer acetate; HCl, hydrochloride; IFN, interferon; i.m. intramuscular; i.v. intravenous; NDC, National Drug Code; s.c. subcutaneous. (DOCX) [file pone.0088472.s001.docx]

**Table S1. NDCs for DMTs of interest.**

| **Drug subclass** | **Generic name** | **Brand name** | **NDC(s)** |
| --- | --- | --- | --- |
| Sphingosine 1-phosphate receptor modulator | Fingolimod HCl, 0.5 mg capsule | Gilenya^®^ | 00078060751 |
| GA | GA for s.c. injection, 20 mg/ml | Copaxone^®^ | 00088115003, 00088115330,68115075030, 68546031730 |
| Monoclonal antibody | Natalizumab for i.v. infusion, 300 mg/15 ml | Tysabri^®^ | 59075073015 |
| IFNs | IFN beta-1a for i.m. injection, 30 µg | Avonex^®^ | 59627000103, 59627000104, 54569443300, 59627000205, 59627000207, 59627000304 |
|  | IFN beta-1a for s.c. injection, 22 or 44 µg/0.5 ml | Rebif^®^ | 44087002203, 44087002201, 44087004403, 44087004401, 44087882201 |
|  | IFN beta-1b for s.c. injection, 0.3 mg | Extavia^®^ | 00078056912, 00078056961 |
|  | IFN beta-1b for s.c. injection, 0.3 mg | Betaseron^®^ | 50419052101, 50419052103, 50419052105, 50419052115, 50419052309, 50419052315, 50419052325, 50419052335 |
