# Supplementary material for: Relapse Rates in Patients with Multiple Sclerosis Switching from Interferon to Fingolimod or Glatiramer Acetate: A US Claims Database Study
Source: PLoS One. 2014 Feb 6;9(2):e88472. doi: 10.1371/journal.pone.0088472 (PMC3916439; doi:10.1371/journal.pone.0088472)
Supplement: Table S2 — Procedural codes for DMTs administered in the clinical setting. DMT, disease-modifying therapy; GA, glatiramer acetate; IFN, interferon; N/A, not applicable. (DOCX) [file pone.0088472.s002.docx]

**Table S2. Procedural codes for DMTs administered in the clinical setting.**

| **DMT** | **Procedural code** |
| --- | --- |
| Fingolimod | N/A |
| GA | Q2010 (terminated 12/31/2003; injection, GA, per dose)  J1595 (injection, GA, 20 mg) |
| Natalizumab | C9126 (terminated 3/31/2005; injection, natalizumab, per 5 mg)  J2323 (injection, natalizumab, 1 mg)  Q4079 (terminated 12/31/2007; injection, natalizumab, 1 mg) |
| IFN beta-1a | Q3025 (injection, IFN beta-1a, 11 µg for intramuscular use)  Q3026 (injection, IFN beta-1a, 11 µg for subcutaneous use)  J1825 (terminated 12/31/2010; injection, IFN beta-1a, 33 µg)  J1826 (injection, IFN beta-1a, 30 µg) |
| IFN beta-1b | J1830 (injection IFN beta-1b, 0.25 mg [code may be used for Medicare when drug is administered under the direct supervision of a physician, not for use when drug is self-administered]) |
